# Investigating human geographic origins using dual-isotope (^87^Sr/^86^Sr, δ^18^O) assignment approaches

**DOI:** 10.1371/journal.pone.0172562

**Published:** 2017-02-21

**Authors:** Jason E. Laffoon, Till F. Sonnemann, Termeh Shafie, Corinne L. Hofman, Ulrik Brandes, Gareth R. Davies

**Affiliations:** 1 Faculty of Earth and Life Sciences, Vrije Universiteit, Amsterdam, The Netherlands; 2 Faculty of Archaeology, Leiden University, Leiden, The Netherlands; 3 Department of Computer & Information Science, University of Konstanz, Konstanz, Germany; Museo delle Civiltà, ITALY

## Abstract

Substantial progress in the application of multiple isotope analyses has greatly improved the ability to identify nonlocal individuals amongst archaeological populations over the past decades. More recently the development of large scale models of spatial isotopic variation (isoscapes) has contributed to improved geographic assignments of human and animal origins. Persistent challenges remain, however, in the accurate identification of individual geographic origins from skeletal isotope data in studies of human (and animal) migration and provenance. In an attempt to develop and test more standardized and quantitative approaches to geographic assignment of individual origins using isotopic data two methods, combining ^87^Sr/^86^Sr and δ^18^O isoscapes, are examined for the Circum-Caribbean region: 1) an Interval approach using a defined range of fixed isotopic variation per location; and 2) a Likelihood assignment approach using univariate and bivariate probability density functions. These two methods are tested with enamel isotope data from a modern sample of known origin from Caracas, Venezuela and further explored with two archaeological samples of unknown origin recovered from Cuba and Trinidad. The results emphasize both the potential and limitation of the different approaches. Validation tests on the known origin sample exclude most areas of the Circum-Caribbean region and correctly highlight Caracas as a possible place of origin with both approaches. The positive validation results clearly demonstrate the overall efficacy of a dual-isotope approach to geoprovenance. The accuracy and precision of geographic assignments may be further improved by better understanding of the relationships between environmental and biological isotope variation; continued development and refinement of relevant isoscapes; and the eventual incorporation of a broader array of isotope proxy data.

## Introduction

Studies investigating the geographic origins of samples of unknown provenance based on comparison with isoscapes (isotope landscape = maps of spatial isotopic variation) [1] have primarily focused on stable isotope measurements (δ^2^H, δ^13^C, δ^15^N) of modern feather samples [2–9]. Although numerous studies [10–15] have also utilized isotope data from modern hair and skeletal materials as geoprovenance indicators in forensic contexts, very few [16–18] have made explicit use of associated isoscapes for these purposes. Similar applications to archaeological research have been lacking to date owing in part to the absence of relevant (bioavailable) large-scale strontium (^87^Sr/^86^Sr) isoscapes for most regions of the world. Furthermore, although the IsoMAP cyber-GIS system [19] is commonly employed for a wide range of geographic assignment applications, it currently lacks functionality for strontium isotopes limiting its utility for many archaeological and forensic provenance studies. To date, archaeological studies have yet to make use of the recent advances in the application of multivariate statistical and probabilistic approaches to isotopic geographic assignment models in wildlife ecology research [7–9,20].

Here we apply and compare two different approaches for utilizing combined strontium (^87^Sr/^86^Sr) and oxygen (δ^18^O) analyses and associated isoscapes for human geoprovenance purposes. The two approaches are applied to dual-isotope dental enamel data of both modern (known origin) and archaeological (unknown origin) samples in the Circum-Caribbean. This region has been chosen as it is one of the few areas of the world for which an accurate spatially-explicit bioavailable ^87^Sr/^86^Sr isoscape has been developed [21,22]. The aim is to develop and test geographic assignment models to estimate origins from skeletal isotope data in the Circum-Caribbean region combining two isotope systems (strontium and oxygen). Longer term goals include improving isotopic geographic assignment methodology for applications in the Circum-Caribbean region and contributing to the further development of isotopic geoprovenance tools in archaeological and forensic studies more generally.

## Materials and methods

### Isotope analyses

Sample preparation and measurements of strontium (^87^Sr/^86^Sr) and oxygen (δ^18^O) isotope compositions were conducted at the Faculty of Earth and Life Sciences, Vrije Universiteit Amsterdam using standard procedures for isotopic analyses of human dental enamel. Details of the analytical procedures (S1 File) are reported elsewhere [22–24]. Oxygen isotope values were obtained from the structural carbonate component of the hydroxyapatite, after chemical pre-treatment of the enamel to minimize the effects of diagenesis, and are reported as delta (δ) values in parts per thousand (‰) relative to the VSMOW scale.

### Enamel samples

Dental enamel is the most highly mineralized substance in the human body (~96% mineral) and is comprised mostly of calcium hydroxyapatite (Ca_10_(PO_4_)_6_(OH)_2_) [25]. A combination of various properties of dental enamel- the fine crystalline structure, avascular nature, and low organic content- prevent substantial remodeling or *in vivo* alteration of the inner core enamel and also make it highly resistant to diagenesis or post-mortem alteration [26]. Therefore, the isotopic signal contained in dental enamel reflects the biogeochemical environment where it formed via the incorporation of elements from consumed food and water during the period of crown mineralization [26]. Enamel of deciduous teeth begins to develop and mineralize *in utero* and is generally completely formed in early infancy [27]. Deciduous molar enamel is thus expected to reflect the combined isotopic contribution of the mother to fetal tissue growth development during pregnancy and also during early infancy if breastfed. Permanent teeth crowns form at different periods throughout childhood. Premolar crowns mineralize from roughly 3–6 years of age [25,27] and thus reflect early childhood dietary inputs. The isotopic signatures of modern dental enamel generally reflect the consumption of local resources but with some possible incorporation of nonlocal resources based on the globalized nature of the food industry.

Dental samples for isotope analyses consist of one modern tooth of known origin from mainland Venezuela, and two archaeological permanent teeth (premolars) from two separate indigenous sites in the insular Caribbean (Table 1). The single modern sample (V1) is a deciduous molar crown of an individual born and raised in Caracas, Venezuela and whose mother is an omnivore and also resided in Caracas (during her pregnancy). This individual was also exclusively breastfed during the first several months of infancy. While we recognize that breastmilk may be enriched in the heavier ^18^O isotope relative to consumed water [28], we have chosen not to apply an offset to correct for this. This decision is based on the difficulty of estimating the relative proportions of the deciduous molar enamel that formed *in utero* relative to the amount that formed during breastfeeding, and also because the reported ^18^O-enrichment of breastmilk is not well characterized being both variable and based on a relatively small sample size (e.g. [28]). Because of the known origin of this individual it is possible to directly test the validity of the geoprovenance approaches discussed herein.

**Table 1:** Sample information and isotope results of test samples.

Sample	Location	Country	Chronology	Element	^87^Sr/^86^Sr	δ^18^O_sc_ ‰
V1	Caracas	Venezuela	Modern	Dec. Molar	0.71013	25.85
F267B	Manzanilla	Trinidad	Pre-colonial	Premolar2	0.72179	28.71
CM72B	El Chorro	Cuba	Early colonial	Premolar1	0.70757	27.14

Two archaeological samples of unknown origin were chosen to directly explore the robustness of these approaches for archaeological applications. One archaeological sample (F267B) is a second premolar of a juvenile (c. 9–11 years of age at death) from the late pre-colonial site of Manzanilla, Trinidad which contains both late Palo Seco and early Arauquinoid cultural materials (dating to c. A.D. 400–900) [29]. This individual was chosen owing to his/her highly elevated ^87^Sr/^86^Sr ratio (0.72179), which is much higher than the measured or predicted ^87^Sr/^86^Sr ranges for the island of Trinidad (0.7083 to 0.7115) or the adjacent Lesser Antilles (c. 0.7055 to 0.7095) and thus was indicative of a possible mainland natal origin [22–23]. Based on archaeological (e.g. lithic ornaments, ceramic styles) and ethnohistoric (documented indigenous oral histories) evidence there were strong connections between communities on Trinidad and coastal South America including migrations of peoples [30].

The second archaeological sample (CM72B) derives from an adult female (c. 18–25 years of age) recovered from the contact/early colonial period (c. A.D. 1450–1600) site of El Chorro de Maíta, Cuba [31]. The ^87^Sr/^86^Sr value of this individual (0.70757) is atypical of the local environment but within the range for Cuba and the Antilles more generally [22–23]. A multidisciplinary study of this individual, however, provided multiple indicators of non-local or exotic origins including non-native: burial practices (prone with a large stone placed over the back of the legs); dental modification; cranial modification; and highly elevated enamel and bone collagen carbon isotope (δ^13^C) values [24,32]. Based on the combined evidence it has been proposed that this individual (CM72B) had origins in the Maya region of Mesoamerica, and that she likely represents one of thousands of individuals who migrated to Cuba during the early colonial period [33]. Owing to the wide range of independent evidence indicative of Mesoamerican origins, this individual was considered ideal to further test the geoprovenance approaches.

### Ethics statement and data availability

All necessary permits were obtained, which complied with all relevant regulations. Permission to study the archaeological specimens was provided via MOUs with the relevant authorities on Trinidad (National Archaeological Committee of Trinidad and Tobago, and the Department of History of the University of the West Indies); and Cuba (Holguin Provincial Monuments Commission, and the National Monuments Commission of Cuba): permit #PEA -1/15. The archaeological specimens analyzed for this study are temporarily curated by the Caribbean Research Group at the Faculty of Archaeology, Leiden University, The Netherlands, and are not publicly deposited and/or accessible by others in a permanent repository.

### Isoscapes

**Fig 1:**
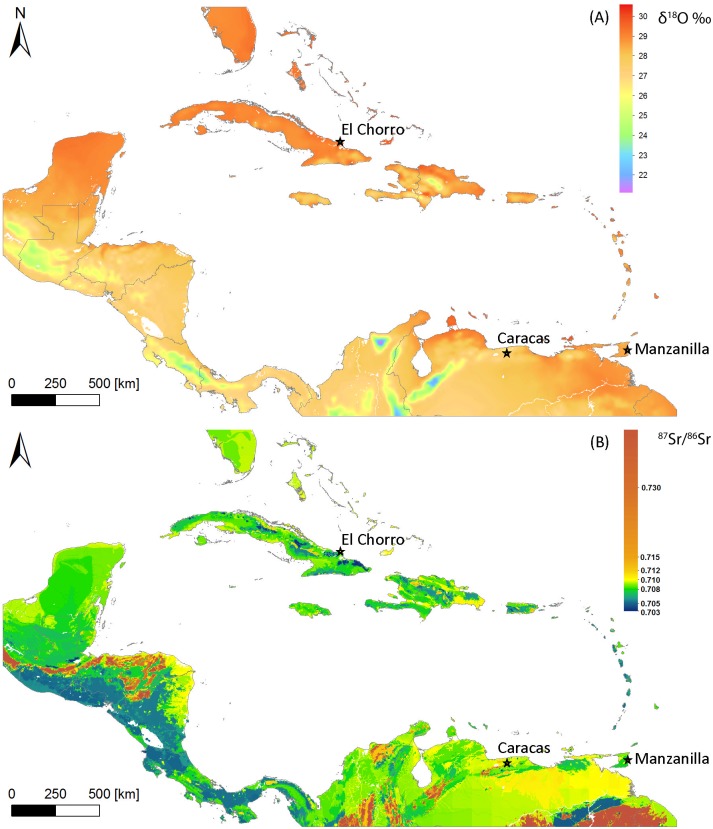
Enamel A) Oxygen (δ^18^O) and B) Strontium (^87^Sr/^86^Sr) isoscapes for the Circum-Caribbean. The enamel δ^18^O isoscape is based on the RCWIP cluster model of the Global Network of Isotopes in precipitation (GNIP) database from Terzer et al. [34–36], and converted to enamel values based on the equation of Chenery et al. [37]. The enamel ^87^Sr/^86^Sr isoscape is modified from the bioavailable multi-source mixing model of Bataille et al. [21].

For the purpose of geo-referencing enamel samples of unknown origin based on measured isotope results we developed *enamel* isoscapes of strontium and oxygen for the Circum-Caribbean (Fig 1). The Caribbean enamel ^87^Sr/^86^Sr isoscape was derived from a previously developed isoscape of bioavailable ^87^Sr/^86^Sr [21] for the region representing a multiple source mixing model of distinct sources of strontium from bedrock weathering and atmospheric aerosols (sea salt and mineral dust). This multi-source model provides spatially explicit estimates of bioavailable ^87^Sr/^86^Sr variation based on estimated relative contributions of Sr from underlying geology, precipitation, and dryfall combined with associated ^87^Sr/^86^Sr values of these three sources [21]. This bioavailable ^87^Sr/^86^Sr isoscape has been tested on large-scale empirical datasets (n = 386) of bioavailable strontium isotope measurements from modern plants, and both archaeological and modern animal samples. The results indicate that this model is highly accurate at predicting biosphere ^87^Sr/^86^Sr for both insular and mainland areas of the Circum-Caribbean with mean absolute error (MAE) between predicted and observed values of 0.00014 and 0.00040, respectively [21]. Because strontium does not undergo substantial trophic level fractionation, it is assumed that the bioavailable ^87^Sr/^86^Sr and human dental enamel ^87^Sr/^86^Sr are highly comparable if not equivalent. The Circum-Caribbean enamel δ^18^O isoscape is derived ultimately from a database of oxygen isotope values (RCWIP_grid_5_13_100_20130502) measured in precipitation by the IAEA from the GNIP (Global Network of Isotopes in Precipitation) project [34], which has been interpolated using the regionalized cluster-based water isotope prediction model (RCWIP) [35–36]. The precipitation oxygen isoscape was converted into an enamel bioapatite (biological apatite) isoscape using a modified version of the conversion equation [δ^18^O_enamel_ = (δ^18^O_precipiation_ + 48.634)/1.590] reported in [37].

In order to combine these two independent isoscapes (^87^Sr/^86^Sr and δ^18^O) it was necessary to conduct various steps of data conversion, reduction, and manipulation. The first step was to create comparable data sets. The higher resolution ^87^Sr/^86^Sr isoscape map of one km^2^ [21] was used as a reference scale. The global precipitation oxygen isotope data set was masked to the size of the Caribbean ^87^Sr/^86^Sr isoscape. The GNIP δ^18^O data is an even GIS grid of points with interpolated values and (modeled) prediction uncertainties, with each point c. 9km distance from another, with no data displayed over water bodies. Compared to continental data, the large water body reduces the number of data points in the Caribbean significantly; some smaller islands lie outside the grid points, and are missing oxygen isotope information at this scale. To create a comparable data set, the oxygen point data set needed to be interpolated into an evenly distributed grid of higher resolution [36]. A land body polygon data set [38] which had been derived from a 10m resolution world coastline data, and included the majority of larger islands in the Caribbean, served as a base map.

**Fig 2:**
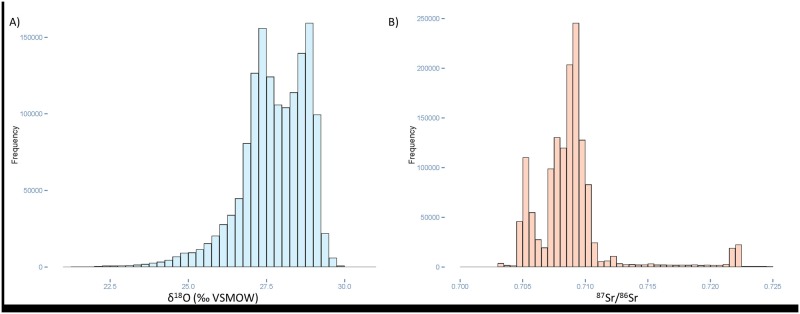
Histograms displaying the overall distribution of δ^18^O and ^87^Sr/^86^Sr values within the Circum-Caribbean enamel isoscapes. Histograms display the distributions of isotope values of individual raster cells (pixels) in the A) δ^18^O; and B) ^87^Sr/^86^Sr isoscapes. The enamel δ^18^O isoscape is based on the RCWIP cluster model of the Global Network of Isotopes in precipitation (GNIP) database from Terzer et al. [34–36], and converted to enamel values based on the equation of Chenery et al. [37]. The enamel ^87^Sr/^86^Sr isoscape is modified from the bioavailable multi-source mixing model of Bataille et al. [21].

The interpolation comprised all point information according to proximity of any land pixel to data points, including data that due to changes in scale appeared to be located offshore next to coastlines. This is achieved using a *cubic spline interpolation* that is designed to produce a smooth data surface in the first derivative, and continuous distribution in the second derivative, both within an interval and at its boundaries [39]. The ArcGIS version, *Interpolation Spline with Barriers* algorithm uses a polynomial that connects all knots, or data points, with original values at each knot location being unchanged. It estimates new values by applying a mathematical function that minimizes overall surface curvature, leading to a smooth surface that (1) passes exactly through the input points, with (2) the barriers honoring discontinuities encoded in both the input barriers and the input point data. The data set was interpolated to a raster of one km resolution. Islands and land areas without values were given the interpolated value from the closest data points on neighboring islands. The interpolated oxygen isotope value was cropped using the earth data polygon. To have comparable data sets without data gaps in different areas of each data set, strontium and oxygen isoscapes, as well as the oxygen error raster dataset, were then subtracted from each other, so each pixel received a value in all data sets (Fig 2).

## Assignment methods

The aim of the assignment approaches presented here is to associate the dual isotope values of a sample with its most likely geographic origin, and to assess the improvement in assigning origins using information from two isotope systems rather than just one. The two main approaches explored for this study are based on assignment to origins of individual raster cells within isoscapes. In this section, we provide details on how individual origins were assigned for three test samples based on a simple Interval Approach using a defined range of fixed isotopic variation for each raster cell; and a Likelihood Approach to probabilistic assignment of geographic origins using univariate and bivariate probability density functions applied to dual isotope results from the same three test samples.

### Interval approach

The interval approach compares the derived enamel sample results with each of the ^87^Sr/^86^Sr and δ^18^O isoscapes for the Caribbean directly. The map values determined for the measured sample have to lie within a given error margin of the measured values (e.g. sample V1: ^87^Sr/^86^Sr = 0.71013, δ^18^O = 25.8), determined through the associated sources of variation [4,40]. The aim of this approach is to determine which (if any) of the raster cells have isotope ranges that overlap with the measured isotope values of the sample(s).

The analytical errors (from the laboratory measurements) are insignificant compared to the expected random variation in isotope values at a given location and the known inaccuracies of the predictive models and uncertainties associated with the isoscape interpolations. For example, the observed inaccuracies of the bioavailable ^87^Sr/^86^Sr isoscape are more than an order of magnitude greater than the typical measurement or analytical error (<0.00001; 1SE) of the enamel ^87^Sr/^86^Sr isotope analyses, while the typical analytical uncertainty of δ^18^O measurements (<0.1‰; 1SD) is also substantially less than the associated interpolation errors of the precipitation δ^18^O isoscape [36]. To define a range of isotope values above and below the mean value provided by the isoscape(s), we have estimated the total error/variance (Ɛ) that includes an estimate of the sum of the analytical error and the population variance (*σ*_j_), and the observed mean absolute error (MAE) of the interpolated isoscapes (σ_M_) [31].

$$\varepsilon =\sigma_{\text{j}}+\sigma_{\text{M}}$$

Variance can be estimated from the (random) deviation of isotope measurements of known-origin samples collected at single sites which encompasses both analytical uncertainty and population variance [4]. For strontium, a constant error was calculated: σ_j_ = 0.0004, based on the average of the standard deviations for all locations (n = 30) within the Antillean bioavailable (archaeological rodent teeth and snail shells, and modern plants) ^87^Sr/^86^Sr dataset (n = 287 samples) [22, nd]. Inaccuracies of the ^87^Sr/^86^Sr isoscape were incorporated by taking the largest MAE between predicted and measured ^87^Sr/^86^Sr in the Circum-Caribbean [31] of σ_M_ = 0.0004; these two errors combined are represented by Ɛ_Sr_ = 0.0008. For oxygen, a constant error based on the average of standard deviations (n = 333 samples) of multiple archaeological human sample populations (n = 12) in the Antilles [24, nd] was calculated: *σ*_*jO*_ = 0.8; to which the mean pixel error (prediction uncertainty); *σ*_*M*_ = 1.2 of the complete Circum-Caribbean δ^18^O isoscape [34–35] was added, resulting in a total error value of Ɛ_O_ = 2.0. However, the δ^18^O isoscape is also associated with a map of prediction uncertainty from the RCWIP model, with interpolated errors varying across the isoscape between raster cells (RCWIP_grid_err_5_13_100_20130502) [34–35], which has to be incorporated into the equation.

*Sr*_*s*_ = Strontium isotope (^87^Sr/^86^Sr) value of sample

*Sr*_*i*_ = Pixel value from ^87^Sr/^86^Sr isoscape map

*O*_*s*_ = Oxygen isotope (δ^18^O) value of sample

*O*_*i*_ = Pixel value from δ^18^O isoscape map

*Ɛ*_*Oi*_ *=* Pixel value from δ^18^O isoscape error variability map

**Fig 3:**
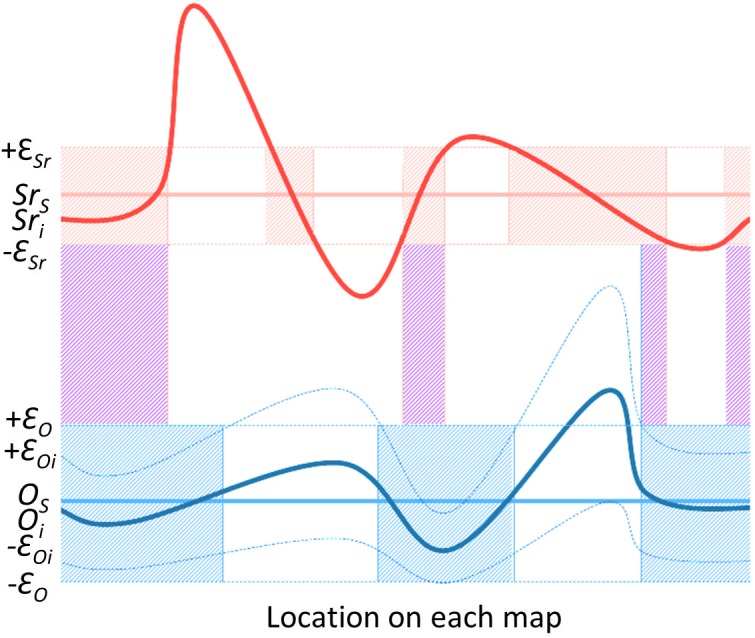
Sketch of the interval approach with constant error for the ^87^Sr/^86^Sr isoscape and a variable error for the δ^18^O isoscapes, and the resulting overlap when comparing both results.

A cell *i* is considered a potential place of origin of a sample *s* if and only if *Sr*_*i*_ is in the range [*Sr*_*s*_ *- Ɛ*_*Sr*_, *Sr*_*s*_ *+ Ɛ*_*Sr*_] and the interval [*O*_*i*_ *- Ɛ*_*Oi*_, *O*_*i*_ *+ Ɛ*_*Oi*_] is fully contained in [*O*_*s*_ *- Ɛ*_*O*_, *O*_*s*_ *+ Ɛ*_*O*_]. The measured enamel isotope data of three test samples were used to conduct geographic assignments to maps of predicted enamel isotope composition (isoscapes) over the area in question using this interval approach as illustrated in Fig 3. Isotope values from enamel samples are compared to this combined isoscape and if both values fall within the range this cell is coded as a one (a potential place of origin) and assigned a color on the corresponding output map. In contrast, raster cells that do not overlap with the measured enamel value for either isotope systems are assigned a score of zero (not a potential place of origin) and assigned a different color on the corresponding output map.

### Likelihood approach

In the likelihood approach the dual-isotope values of the test sample(s) are used to assess the likelihood of specific geographic origin by calculating a spatially explicit normal probability density function (e.g. a probability density surface). The method refines the filtering procedure introduced in the interval approach section by either avoiding thresholding or by indicating how stable the filtering is. Moreover, this approach further demonstrates that using information from two isotope systems rather than one leads to an improvement when assigning plausible origins. Let *X* denote the random isotope variable with values *x* following probability distributions *f*(*x*|*c*) for each raster cell *c* = 1,…, *C*. The most likely origin for a test sample x* is determined by evaluating the likelihood at each cell *c*, and the largest *f*(x*|*c*) is the most likely origin point which generates our observed value x*. It is reasonable and common to assume that *X* is normally distributed with parameters *μ*_*c*_ and *σ*^2^ [2,9]. The *μ*_*c*_ are equal to the expected values at each raster cell extracted from the isoscapes.

Given the observed values of each isotope variable, the univariate standard normal distribution is used for calculating the likelihoods. The standardization of the normal distribution is given by

$$z^{*}=\;\frac{x^{*}-\mu_{c}}{\sigma }$$

with probability density function

$$f\left(z^{*}|c\right)=\frac{1}{\sqrt{2\pi }}\text{exp}\left(-\frac{z^{*2}}{2}\right)$$

and where z* is changed according to whether the observed ^87^Sr/^86^Sr and δ^18^O value of our test samples are considered. Similarly, the bivariate standard normal distribution is used to assess the joint likelihood of two isotope variables. Under the assumption of independence between the two variables, the bivariate probability density function simplifies to

$$f\left(z_{O}^{*},z_{Sr}^{*}\;|c\right)=\frac{1}{2\pi }\text{exp}\left(-\frac{z_{O}^{*2}+z_{Sr}^{*2}}{2}\right)$$

where z*_O_ and z*_Sr_ are the observed values of the test samples. This joint function is the product of the marginal functions based on each isotope system, and yields high likelihood when both the marginal densities are also high.

## Results

Two main approaches for investigating geographic origins based on measured ^87^Sr/^86^Sr and δ^18^O values in dental enamel were developed, tested, and compared: 1) an Interval Approach [16]; and 2) a Likelihood Approach based on normal probability density functions [9]. The results of each approach are presented and compared in this section.

### Interval approach

**Fig 4:**
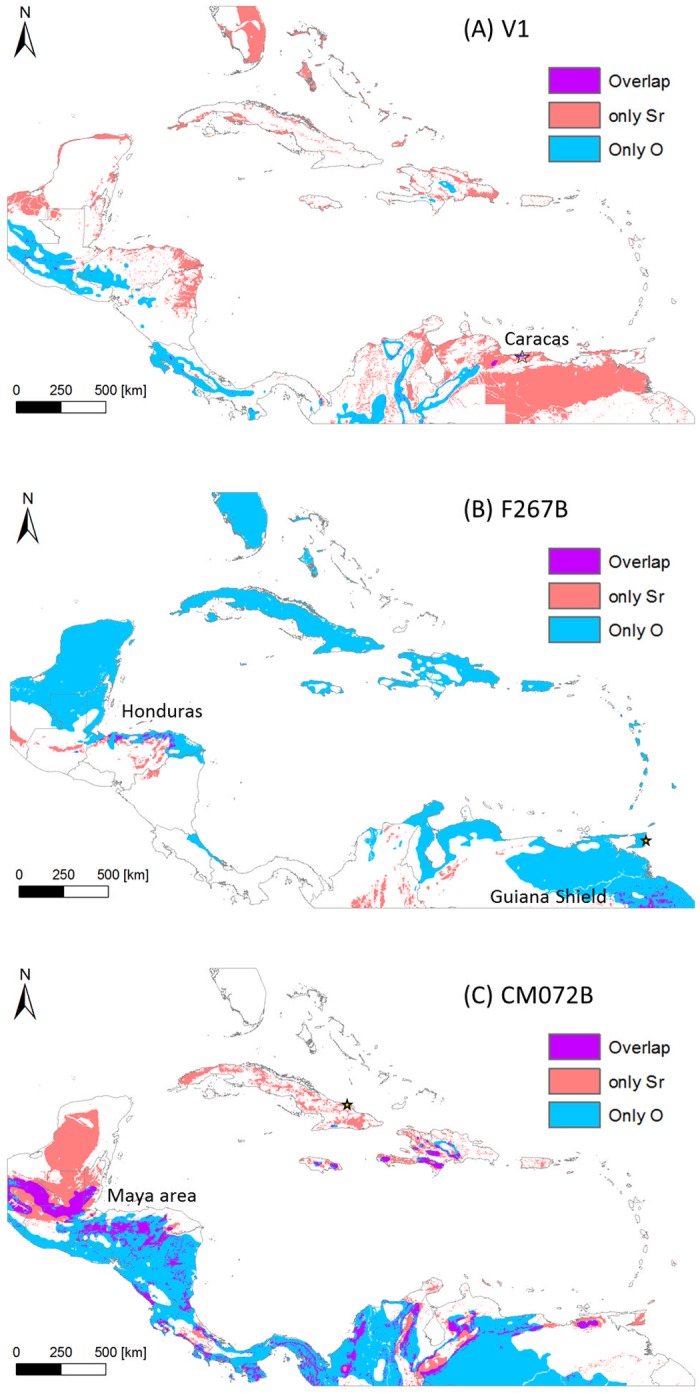
Predicted natal origin of test samples based on the interval approach. If the sample’s isotope value(s) is within the range defined by the mean ±2σ for a given raster cell, the estimated possible natal origin is highlighted in red (^87^Sr/^86^Sr), blue (δ^18^O), or purple (both ^87^Sr/^86^Sr and δ^18^O).

The results of the interval approach applied to the test samples are displayed in Fig 4. Sample V1 is a modern tooth of an individual with known (natal) origin from Caracas, Venezuela. The output of the interval approach applied to this individual’s measured isotope values not only eliminates the vast majority of locations within the Circum-Caribbean as possible locations of origin but also correctly identifies Caracas as a place of possible origin. Similarly, for sample F267B (Manzanilla, Trinidad) most of the overall map area is excluded as a possible natal origin for this individual, and only a few small clusters are highlighted as possible places of origin, including large but non-discrete portions of the Guiana Shield Region of north-eastern South America (south of the Orinoco River), as well as areas in northern Central America (in northern Honduras). In contrast to sample V1, for sample F267B, the ^87^Sr/^86^Sr data provided much more geographic discrimination than the δ^18^O data. Likewise, for sample CM72B (El Chorro de Maíta, Cuba) the ^87^Sr/^86^Sr data excluded a larger area than the δ^18^O data and combining both isotopes provided the narrowest range of possible origins. The proportion of the map highlighted as possible origins for CM72B was larger and more spatially dispersed than the other two test samples. Additionally, the proposed origin in the Maya area is consistent with the results of the interval approach that indicated large swaths of this region as possible places of origin.

These successful but variable results validate the interval approach in general, and also highlight both the potential (the capacity to exclude many potential places of origin) and limitation (inability to distinguish locations with similar ranges of isotope values for both strontium and oxygen) of this approach. In addition, as previously noted [4], one of the main critiques of these types of assignment is that they are inherently binary, assigning each location on a map into either the category of possible or not possible. As such, they are not only simplistic and potentially misleading but also do not permit explorations or comparisons of relative likelihood or probability across the region of interest.

### Likelihood approach

**Fig 5:**
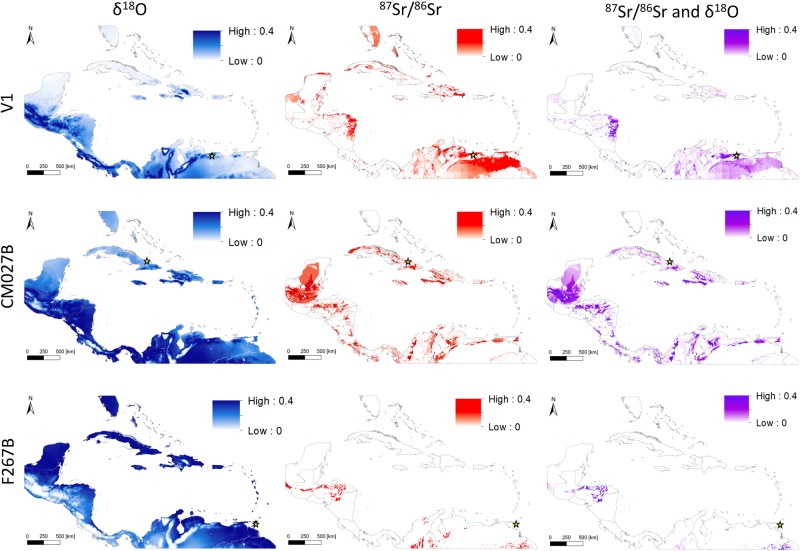
Probability density surfaces for test samples based on spatially explicit univariate (δ^18^O), (^87^Sr/^86^Sr); and bivariate (^87^Sr/^86^Sr and δ^18^O) normal probability density functions. Note: The values plotted are the calculated probability density values and not probabilities (i.e. they do not sum to unity).

The results of the likelihood approach using univariate and bivariate normal probability density functions (pdf) applied to the isotope values of the test samples are displayed in Fig 5. A comparison of the results of the univariate pdf and bivariate pdf analyses is especially illuminating for sample V1, as the bivariate normal pdf clearly provided improved resolution compared to either the univariate ^87^Sr/^86^Sr or δ^18^O pdf. Perhaps not surprisingly, the results for sample V1 using the bivariate normal pdf approach are very comparable to the results of the interval approach, and indicate a very low likelihood of origin for most locations within the map area and correctly indicate Caracas as a place of origin with high likelihood. Similarly, for sample F267B, the bivariate normal pdf provides more geographic discrimination than either univariate pdf, and both the interval and bivariate normal pdf approaches provide very similar results in terms of the general areas that are excluded and the areas highlighted as possible origins (e.g. primarily clustered in the Guiana Shield Region of north-eastern South America; and in and around Honduras). In contrast to the other test samples, the results of the pdf analyses for sample CM72B were quite different. For both of the univariate pdfs, relatively smaller areas of the map were left unshaded and correspondingly much more of the overall map was highlighted as places of high likelihood of origins. This suggests that individual CM72B’s combined isotope values are in general much more common within the bounds of the Circum-Caribbean region defined by our isoscapes. As such, the bivariate normal pdf generated probability density surfaces for CM72B highlighting much of the overall map as possible areas of origin and many different locations as having high likelihood of childhood origin. Nonetheless, it is noteworthy that the previously proposed place of origin in the Maya region (namely Belize) based on other lines of evidence for sample CM72B is also highlighted as having high likelihood based on the result of the bivariate normal pdf.

## Discussion

The interval approach applied to the three test samples in this study provided simple, apparently accurate, and straightforward output results concerning possible place of origin. The primary constraints of this approach, however, are that it is potentially a misleading approach in that it incorporates strict and finite cutoff limits or ranges of isotope variation for each isoscape raster cell. Secondly, the output results are a binary division into two simple outcomes: possible/not possible and there is a high degree of uncertainty concerning the appropriate cut-off between the two, i.e. which sources of error (or variation) are most appropriate for each isotope system [4]. To some extent the incorporation of associated errors is somewhat *ad hoc* and slightly different for the two isotope systems.

The likelihood approach (both the univariate and bivariate normal pdfs) represents a distinct improvement over both the traditional method of simple visual comparison of individual isotope results to one or more isoscapes, and the interval approach. The most important advantage of the likelihood method relative to the interval method is that whereas the latter treats all possible origins as equally plausible, the latter provides output results that display spatial variation in relative ‘likelihood’ across the map area. The high degree of specificity and the high likelihood assigned to the area of Caracas based on the isotope values of the known origin sample (V1) provide strong validation that the bvnpdf method works well as a geoprovenance tool. Further work will be required to replicate these results on more known origin samples that represent a wider range of geographic origins and combinations of isotope values.

It is self-evident, but worth explicitly noting, that the geographic assignment methods discussed herein are only as reliable as the underlying isoscapes upon which they are based. Given the relatively minor contribution of analytical errors to overall variance (including population variance; prediction uncertainty) for the isotope systems discussed herein, the overall success of these geoprovenance methods would be most readily advanced by improving the underlying isoscapes.

For ^87^Sr/^86^Sr, both the extent and nature of population variance (intra-population or within location) should be more thoroughly and systematically addressed, and although treated as a constant in this study, it is likely to be spatially variable. For example, calculations of the mean (absolute or relative) difference between predicted and measured bioavailable ^87^Sr/^86^Sr values in the Circum-Caribbean indicate that both the MAE and RAE are overall quite small [21] but they are also variable across the overall study area (i.e. between different locations and regions therein). Furthermore, for many locations there is simply insufficient empirical (bioavailable) ^87^Sr/^86^Sr data available, particularly for inland regions of northern South America, so currently the accuracy of the extant ^87^Sr/^86^Sr isoscape across the entire region is difficult to assess. This points to the need for both larger and more geographically and geologically representative data bases of bioavailable ^87^Sr/^86^Sr for the Circum-Caribbean (and elsewhere) in addition to further refinement to the process-based isoscape models themselves. Recent research applying similar predictive modelling approaches to other macro-regional settings (the continental US) [41] demonstrates that such models can accurately predict the ^87^Sr/^86^Sr of mammalian skeletal tissues, providing further confidence in the utility of such models. Nonetheless, we regard the ^87^Sr/^86^Sr isotope data, in general, as much more reliable and informative than δ^18^O data at least for the Circum-Caribbean region.

The δ^18^O data and isoscape suffer from the same issues as discussed for ^87^Sr/^86^Sr, but have additional sources of variation including the issue of time or chronological variation in precipitation δ^18^O and thus associated bioapatite δ^18^O (e.g. climate change). There is some evidence, however, that temporal variation in precipitation δ^18^O in the Caribbean during the late pre-colonial/early colonial periods were minimal compared to spatial δ^18^O variations [42–44]. Nonetheless, more research is required to elucidate the extent of climatic change and other sources of stable isotope variation over the time period in question as the δ^18^O isoscape used herein is based on modern data generated primarily over the second half of the twentieth century. As such it can be questioned to what extent this dataset can be utilized on archaeological samples and for how far back in time the dataset is useful in this regard. Other complications concerning the interpretation of skeletal δ^18^O data are the unknown effects of multiple biological (pathology, physiology) and biocultural processes (breastfeeding/weaning, brewing, boiling) [45]. Additionally, some debate exists concerning the conversion between skeletal δ^18^O and drinking water δ^18^O, and whether the same conversion equation is appropriate for all populations, regions, and environments [37,46]. A further caveat is that unlike similar geographic assignment approaches (e.g. the sourcing of wild animals with generally known or observable ranges) humans can, in principle anyway, originate from almost anywhere, including areas outside of the geographical limits of the associated maps/isoscapes.

An obvious possible improvement to the geoprovenance approaches presented here is the addition of additional isotopic systems, such as lead (Pb) isotopes. Owing to the fact that there are multiple non-stable isotopes of Pb, the inclusion of Pb isotope data actually offers the opportunity to contribute multiple new variables (albeit not independent). While the technology and methodology (chemistry and mass spectrometry) for Pb isotope analyses of skeletal materials (teeth specifically) already exist [47], bioavailable Pb isoscapes have not yet been widely developed. The creation of Pb isoscapes is also somewhat problematic because of the dominance of anthropogenic Pb in modern and many historical (post-metallurgy) eras [47]. Thus, forensic applications would likely require a different (anthropogenic) Pb isoscape than archaeological applications. It should be noted that based on published Pb isotope data (e.g. the GEOROC database) [48] it may be possible to make a 1^st^ order approximation of a bioavailable Pb isoscape at least for parts of the Circum-Caribbean [49] and other areas can be interpolated until such time that more empirical data are generated.

Additionally, substantial archaeological stable isotope data sets (comprising collagen carbon and nitrogen, and apatite carbon) have already been generated for much of the Circum-Caribbean. Although the Caribbean stable isotope data display clear geographical structure [24,50–52] these data are not readily amenable to spatial interpolation (e.g. as isoscapes) at least not for humans, as has been done for similar stable isotope data from various animals in related ecological applications [7,9]. As such, multivariate statistical approaches such as cluster analysis and discriminant function analysis, may prove fruitful for geoprovenance studies in many regions including the Caribbean [15,53]. Furthermore, despite the global nature of the modern food economy, successful applications of multi-isotope provenance studies in forensic research [12–18] demonstrates the utility of these methods, despite the potential complications introduced by the consumption of imported foods.

Avenues for future research include further refinement of the extant ^87^Sr/^86^Sr and δ^18^O isoscapes for the Circum-Caribbean, especially in terms of testing spatial (and temporal) variability in deviations between modelled and measured isotopes values. More isotope data from known origin individuals from the study area would greatly contribute to the further validation of the methods explored in this study. Large-scale multiple isotope data sets (strontium, oxygen, carbon, nitrogen, and lead) for the Circum-Caribbean region can be integrated into a broader multi-variate statistical framework for geoprovenance research. Lastly, the incorporation of a wide range of prior information concerning possible geographic origins: such as environmental, settlement, genetic, morphological, or material culture data into Bayesian approaches [4–8] offers enormous potential for building upon the methods investigated herein.

## Supporting information

S1 File Isotope methods and procedures. (DOCX)
