# Supplementary material for: Investigating human geographic origins using dual-isotope (87Sr/86Sr, δ18O) assignment approaches
Source: PLoS One. 2017 Feb 21;12(2):e0172562. doi: 10.1371/journal.pone.0172562 (PMC5319690; doi:10.1371/journal.pone.0172562)
Supplement: S1 File — (DOCX) [file pone.0172562.s001.docx]

**S1: Isotope Methods and Procedures**

Sample preparation and measurements of strontium (^87^Sr/^86^Sr) and oxygen (δ^18^O) isotope compositions were conducted at the Faculty of Earth and Life Sciences, Vrije Universiteit, Amsterdam under clean lab conditions in designated facilities. Dental samples were mechanically cleaned with a diamond-tipped abrading bit attached to a dental drill (Dremel) and were subsequently sonicated in ultra-pure water (Milli-Q H_2_O). The outer surface of the tooth crown was then removed with a pre-cleaned diamond tipped drill bit and approximately 2-4 mg of inner (core) enamel powder was sampled for isotopic analyses. Powdered enamel was then soaked for 24hours in 2.5% bleach (NaOCl), rinsed to neutral, soaked for 4h in Ca-acetate buffered (pH 4.75) 1.0 M acetic acid (CH_3_COOH), rinsed again, and dried.

For strontium isotope analyses, 1-2mg of enamel was dissolved with 0.5mL 3N HNO_3_. The Sr fraction of the dissolved samples was separated via a low-blank, column chromatography using Sr-spec resin (Eichrom). Purified Sr fractions were loaded onto pre-cleaned Re filaments using a TaCl_5_ activator. Strontium isotope ratios were measured on a Thermo Finnigan MAT262 TIMS (thermal ionization mass spectrometer) using a static multi-collection routine for a minimum of 60 scans. Results were corrected for mass fractionation using an exponential law and an ^86^Sr/^88^Sr ratio of 0.1194. The international NBS987 standard was analyzed to monitor system performance. The long term average ^87^Sr/^86^Sr ratio of NBS987 is 0.71024 ± 0.00004(2σ). The ^87^Sr/^86^Sr isotopes of the measured samples have been normalized to the NBS987 value of 0.710240. Total procedural blanks are consistently <100pg, which is negligible relative to the typical Sr content of measured samples (>100ng).

For oxygen isotope analyses, 0.5-1mg of enamel was weighed into pre-cleaned glass sampling tubes, loaded into a hotblock at 45°C for 24h and dissolved with 100% orthophosphoric acid (H_3_PO_4_). Oxygen isotope compositions were analyzed using a Delta plus IRMS (isotope ratio mass spectrometer) coupled to a GasBench II. Oxygen isotope values are reported as delta (δ) values in units of per mil (‰) normalized to the PDB scale using an in-house carbonate reference material (VICS) calibrated against certified reference materials (NBS19 and LSVEC). The δ^18^O values have been converted to the VSMOW scale using the published conversion equation (δ^18^O_SMOW_ = 1.03091 ∗ δ^18^O_PDB_ + 30.91) of Coplen (1988). The international IAEA-CO1 standard was analyzed to monitor instrument performance. The reproducibility of IAEA-CO1 during the analytical session was ±0.16‰ (1σ) for δ^18^O.

**Reference**

Coplen TB. Normalization of oxygen and hydrogen isotope data. Chemical Geology: Isotope Geoscience section. 1988; 72(4):293-297.
